# The effect of specimen processing time on HER2 expression in gastric cancer and esophagogastric junction cancer: a single-center retrospective observational study

**DOI:** 10.1186/s12885-023-11148-y

**Published:** 2023-07-11

**Authors:** Yoshitomo Yanagimoto, Hiroshi Imamura, Shiro Adachi, Kazuki Odagiri, Tomono Kawase, Masafumi Yamashita, Hiroshi Takeyama, Yozo Suzuki, Masakazu Ikenaga, Junzo Shimizu, Naohiro Tomita, Keizo Dono

**Affiliations:** 1 Department of Surgery, Toyonaka Municipal Hospital, 4-14-1 Shimahara-Cho, Toyonaka, Osaka 560-8565 Japan; 2 Department Diagnostic Pathology, Toyonaka Municipal Hospital, 4-14-1 Shimahara-Cho, Toyonaka, Osaka 560-8565 Japan

**Keywords:** HER2 expression, Gastric cancer, Esophagogastric junction cancer

## Abstract

**Background:**

Recent developments in the field of companion diagnosis and molecular-targeting therapeutic agents have helped in developing treatments targeting human epidermal growth factor receptor 2 (HER2) in gastric cancer (GC) and esophagogastric junction cancer (EGJC), and the importance of accurate diagnosis of HER2 expression is increasing. However, the HER2-positivity rate significantly differs among reports in GC and EGJC, and factors that affect HER2-positivity require elucidation.

**Methods:**

The present study retrospectively examined factors related to HER2-positivity in a single institution, including age, sex, body mass index, the American Society of Anesthesiologists physical status, tumor information, and surgery information, including time to specimen processing.

**Results:**

Our study included 165 patients tested for HER2 using GC and EGJC surgery specimens among the 1,320 patients who underwent gastrectomy from January 2007 to June 2022. In total, 35 (21.2%) and 130 (78.8%) patients were HER2-positive and -negative, respectively. Multivariate analysis revealed that intestinal type (odds ratio [OR]: 3.41, 95% confidence interval [CI]: 1.44–8.09, *p* = 0.005), pM1 (OR: 3.99, 95% CI: 1.51–10.55, *p* = 0.005), and time to specimen processing of < 120 min (OR: 2.65, 95% CI: 1.01–6.98, *p* = 0.049) were independent factors that affected HER2-positivity.

**Conclusions:**

The outcomes of the present study indicated that intestinal type, pM, and time to specimen processing are important factors affecting HER2-positive rates in GC and EGJC. Therefore, the risk of false-negative HER2 results may be reduced by decreasing the time required to process the resected specimen. Additionally, accurate diagnosis of HER2 expression may increase the opportunity to administer molecular-targeted drugs that can expect therapeutic effects to patients appropriately.

**Trail registration:**

Retrospectively registered.

## Background

Human epidermal growth factor receptor 2 (HER2)—an oncogene—has a structure similar to the epidermal growth factor receptor gene. HER2 is expressed in many organs, including the breasts, gastrointestinal tract, kidneys, and heart [1–3]. The HER2 gene coding HER2 protein is a receptor-type protein that goes through the cell membrane, is activated by the tyrosine residue of phosphorylation, and is involved in cell proliferation mediated by RAS/RAF signaling pathways and apoptosis suppression [1–3].

In gastric cancer (GC) and esophagogastric junction cancer (EGJC), immunohistochemistry (IHC) 3 + or IHC 2 + in situ hybridization-positivity indicate HER2-positivity [4, 5]. The relationship between HER2 protein overexpression and/or gene amplification and prognosis in GC and EGJC have been described in several reports; however, studies have reported poor prognosis [6–9], absence of a relationship with prognosis [10, 11], and good prognosis [10, 12], and the assessment of which is controversial. However, developments in recent years have been underway on molecular-targeting therapeutic drugs that target HER2 proteins. The ToGa [13] and DESTINY-Gastric01 trials [14] were conducted on patients with unresectable advanced, recurrent HER2-positive GC and EGJC. In these trials, the group administered Trastuzumab in combination with chemotherapy and Trastuzumab deruxtecan showed a significant improvement in overall survival compared with the group that was administered only chemotherapy. These results show that the strategy using HER2-targeting therapeutic drugs based on expression profiles of HER2 for unresectable advanced, recurrent GC and EGJC is currently adapted [15]. Therefore, HER2 expression should be accurately diagnosed for the treatment strategy of GC and EGJC to provide an opportunity for molecular-targeted drug administration that can appropriately expect therapeutic effects in patients.

The guidelines of the College of American Pathologists, the American Society for Clinical Pathology, the American Society of Clinical Oncology [4], and the Japanese Guidelines for HER2 Testing in Breast Cancer/Gastric Cancer [5] report methods to appropriately handle pathohistological specimens used for HER2 diagnosis. However, the HER2-positivity rate regarding GC and EGJC is 6.0%–29.5%, with a significant difference depending on the report [11, 13, 16–20]. Formalin-fixed paraffin-embedded (FFPE) specimens are often used for HER2 diagnosis; however, the diagnosis accuracy is affected by the “preformalin fixation process” (i.e., the warm ischemic time from cessation of tissue blood flow to resection and cold ischemic time from resection to formalin fixation), the “formalin fixation conditions” (i.e., formalin concentration and pH, and formalin fixation time), and the “postformalin fixation process” (i.e., the decalcification processing, and reagent and FFPE preservation conditions) [21–28]. Among these, the warm and cold ischemic times are important factors involving clinicians (surgeons).

In the most recent Japanese Classification of Gastric Carcinoma, 3rd ed. [29], formalin fixation within 2 h of specimen extraction is recommended. However, the rationale for these recommendations related to handling the pathohistological specimens used for HER2 is based on the results of studies that predominantly used breast cancer specimens. In GC and EGJC, the relationship between HER2-positivity and the time to process surgical specimens has not been elucidated. This study aimed to examine the factors involved in the HER2 positivity of GC and EGJC and to obtain knowledge about the appropriate handling of surgical specimens.

## Methods

### Study design

We included patients who underwent gastrectomy for GC and EGJC at our hospital from January 2007 to June 2022. The present trial was conducted in accordance with the regulations of the Declaration of Helsinki. The study obtained approval from the institutional review board of Toyonaka Municipal Hospital (protocol ID: 20,220,905), and informed consent was obtained before surgery from all patients. Patient data were retrospectively analyzed using the database accumulated prospectively. Patient background included age, sex, body mass index, and American Society of Anesthesiologists physical status. Tumor information included occupied site, histological type, pT factor, pN factor, pM factor, and HER2 score based on the Japanese Classification of Gastric Carcinoma, 15th ed. [29]. Further, tumors were staged according to the Dukes-MAC-like staging system [30]. Surgery information included surgical approach, gastrectomy procedure, surgery time, and time to specimen processing (defined as the time from complete stomach resection to the start of specimen processing). Study inclusion criteria were patients diagnosed histologically as adenocarcinoma of the stomach or gastroesophageal junction and evaluated for HER2 expression in the resected primary tumor specimens. Exclusion criteria were patients evaluated for HER2 expression in biopsy specimens, in tissue specimens from metastatic sites other than the primary tumor, and with insufficient clinical records. The analysis included cases that underwent primary tumor resection for prognosis improvement or symptom relief, such as passage disorder or bleeding, even if they had an M factor. Cases with M1 factor and R1 (microscopic cancer residue) or R2 (macroscopic cancer residue) postoperatively evaluated HER2 expression simultaneously as the surgery ended. HER2 expression was evaluated upon recurrence in cases where recurrence occurred during the postoperative course of radical resection.

### HER2 diagnosis

The tissue was kept in a cooling box (4 °C) immediately after resection. The resected tissue was divided into stomach and lymph nodes by the surgeon; the stomach was then mounted on fixation plates and fixed with fresh 10% Formalin neutral buffer solution without delay. The time for formalin fixation was 6–72 h. Paraffin embedding was performed using the general histological method. For each FFPE block, two sliced or 4–5-μm sections were created, and treated with hematoxylin eosin staining and IHC. In the event of IHC 2 + results, an additional slice was created to perform fluorescence in situ hybridization (FISH). HER2 diagnosis was established on the basis of the Japanese Guidelines for HER2 Testing in Breast Cancer/Gastric Cancer, 2nd ed. [15]. Tissue sections were prepared as mentioned below in accordance with the procedures of the manufacturer. IHC was performed using Pathway anti-HER2 rabbit monoclonal primary antibodies (Roche Diagnostics, Inc., Tokyo, Japan), and FISH was performed using FISH PathVysion HER-2 DNA probe kit (Abbott Japan, Tokyo, Japan). HER2-positivity was defined as IHC 3 + or IHC 2 + and FISH positive. The definitions of IHC scoring criteria and FISH positivity are as follows: IHC score of 0 indicates the absence of staining or a cell membrane staining of < 10% infiltrating cells. An IHC score of 1 + indicates weak cell membrane staining of ≥ 10%, and staining of some cell membrane regardless of the staining intensity. An IHC score of 2 + indicates weak-to-moderate cell membrane staining in ≥ 10% of infiltrating tumor cells. An IHC score of 3 + indicates intense cell membrane staining in ≥ 10% of infiltrating tumor cells. In the event of IHC 2 + , to determine FISH positivity, the fluorescence signal ratio of HER was measured. The ratio of the total number of HER2 signals to the total number of CEP17 signals (HER2/CEP17) was calculated in 20 cancer cells, and a HER2/CEP17 ratio of < 2.0 was defined as gene amplification negativity (FISH-negativity), and that of ≥ 2.0 was defined as gene amplification positive (FISH-positive). In cases when the HER2/CEP17 ratio was > 1.8 and < 2.2, an additional 20 cells were counted and the ratio was determined based on all 40 cells.

### Statistical analysis

Intergroup analyses were performed using the Mann–Whitney U test and Fisher’s exact test. To evaluate independent risk factors of HER2-positivity, we conducted univariate and multivariate analyses to calculate odds ratio (OR) and 95% confidence interval (CI). All continuous variables are presented as median and range. *P* values were two-sided, and *p* < 0.05 indicated statistical significance. Statistical analyses were performed using JMP 10 (SAS Institute, Cary, NC, USA).

## Results

**Fig. 1:**
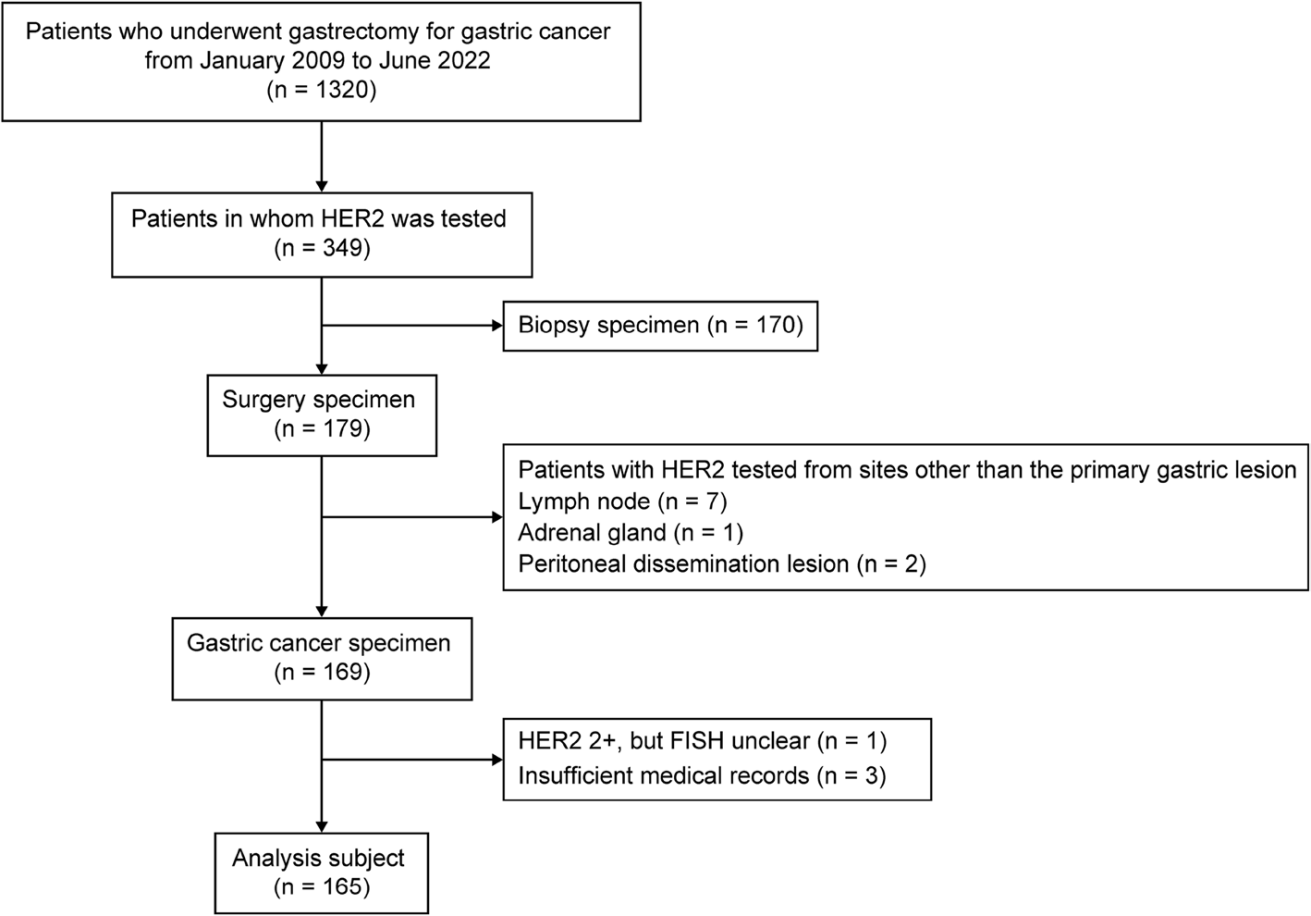
Flow diagram of patient selection. Clinical analysis of HER2 expression during gastrectomy for gastric cancer patients: a retrospective cohort study of 1320 patients

Among the 1,320 patients who underwent gastrectomy for GC and EGJC at our hospital from January 2007 to June 2022 based on the database accumulated prospectively, we included 349 patients who were tested for HER2 expression. Of these, we excluded 170 patients for whom biopsy specimens were used for testing HER2 and included 179 patients for whom surgical specimens were used. Among these surgical specimens, we excluded the specimens of the lymph nodes in 7 patients, those of peritoneal dissemination nodules in 2 patients, and those of the adrenal gland in 1 patient. Of the remaining GC specimens of 169 patients, we excluded those of 3 patients with insufficient medical records, and 1 patient with HER2 IHC2 + but unknown FISH results, and thus 165 patients were included in our study (Fig. 1).

Patients’ background according to HER2 in the present study is presented in Table 1. Of the 165 subjects, 35 patients were HER2 positive (HER2-positive group) (21.2%) and 130 patients were HER2 negative (HER2-negative group) (78.8%). The breakdown was IHC3 + for 21 patients (12.7%), IHC2 + and FISH-positive for 14 patients (8.5%) IHC2 + and FISH-negative for 22 patients (13.3%), IHC1 + for 34 patients (20.6%), and IHC0 for 74 patients (44.8%). The HER2-negative and –positive groups consisted of 17 and 47 cases with cancer residue due to M1 or R1/2 and evaluated HER2 expression at the end of the surgery, respectively. The HER2-negative and –positive groups demonstrated 18 and 83 cases where recurrence was observed during the postoperative course, and HER2 expression was evaluated, respectively (*P* = 0.181). Comparing the HER2-positive group and the HER2-negative group revealed that the significantly common histological type was the intestinal type in the HER2-positive group (HER2-positive group vs. negative group: 71.4% vs. 42.3%, *p* = 0.002), and pM1 was common (31.4% vs. 14.6%, *p* = 0.030). The univariate analysis revealed that intestinal type (OR: 3.41, 95% CI: 1.51–7.68, *p* = 0.003), pM1 (OR: 2.68, 95% CI: 1.12–6.35, *p* = 0.025), and time to specimen processing of < 120 min (OR: 2.63, 95% CI: 1.09–6.67, *p* = 0.033) were factors that affect HER2-positivity. Furthermore, the multivariate analysis revealed that intestinal type (OR: 3.41, 95% CI: 1.44–8.09, *p* = 0.005), pM1 (OR: 3.99, 95% CI: 1.51–10.55, *p* = 0.005), and time to specimen processing of < 120 min (OR: 2.65, 95% CI: 1.01–6.98, *p* = 0.049) were independent factors that affect HER2-positivity (Table 2).

**Table 1:** Patients’ background

	HER2 +	HER2 −	
	(*n* = 35)	(*n* = 130)	P
HER2 score			
IHC 3 +	21 (60.0)	0 (0)	< 0.001
IHC 2 + , FISH +	14 (40.0)	0 (0)	
IHC 2 + , FISH −	0 (0)	22 (16.9)	
IHC 1 +	0 (0)	34 (26.2)	
IHC 0	0 (0)	74 (56.9)	
Age (years)	74 (36–87)	74 (29–91)	0.788
Sex			
Female	11 (31.4)	40 (30.8)	0.940
Male	24 (68.6)	90 (69.2)	
BMI	23.0 (17.4–27.7)	21.7 (14.9–30.1)	0.199
Occupied site			
L: Antrum	14 (40.0)	47 (36.2)	0.481
M: Body	14 (40.0)	44 (33.8)	
U: Upper portion	7 (20.0)	39 (30.0)	
Histological site			
Intestinal tube type	25 (71.4)	55 (42.3)	0.002
Diffuse type	10 (28.6)	75 (57.7)	
ASA			
1	2 (5.7)	16 (12.3)	0.587
2	24 (68.6)	81 (62.3)	
3	9 (25.7)	32 (24.6)	
4	0 (0)	1 (0.8)	
Approach			
MIS	14 (40.0)	42 (32.3)	0.398
Open	21 (60.0)	88 (67.7)	
Procedural type			
DG: Distal gastrectomy	22 (62.9)	69 (53.1)	0.638
PG: proximal gastrectomy	2 (5.7)	17 (13.1)	
TG: Total gastrectomy	10 (28.6)	40 (30.8)	
OT: Other	1 (2.9)	4 (3.0)	
pT			
T1	2 (5.7)	9 (6.9)	0.573
T2	4 (11.4)	13 (10.0)	
T3	14 (40.0)	37 (28.4)	
T4	15 (42.9)	71 (54.62)	
pN			
N0	5 (14.3)	15 (11.5)	0.315
N1	5 (14.3)	30 (23.1)	
N2	10 (28.6)	21 (16.2)	
N3	15 (42.9)	64 (49.2)	
pM			
M0	24 (68.6)	111 (85.4)	0.030
M1	11 (31.4)	19 (14.6)	
Dukes-MAC-like stage			
A1/2	2	9	0.565
B1/2	4	13	
C1/2	14	37	
D	15	71	
Surgery time (min)	303 (176–554)	316 (96–668)	0.576
Time to specimen processing (min)	100 (49–190)	107 (34–477)	0.172

Median (minimum–maximum) or, (%)

*IHC* Immunohistochemistry, *FISH* Fluorescence in situ hybridization, *BMI* Body mass index (kg/m^2^), *ASA* American Society of Anesthesiologists physical status, *MIS* Minimal invasive surgery

**Table 2:** Factors related to HER2-positivity, univariate, and multivariate analysis

		Univariate analysis		Multivariate analysis	
		OR (95% CI)	P	OR (95% CI)	P
Age	75 ≤ vs. < 75	0.82 (0.39–1.75)	0.611		
Sex	Male vs. female	0.94 (0.43–2.17)	0.940		
Occupied site	U vs. M, L	0.58 (0.23–1.49)	0.245		
Histological type	Intestinal tube type vs. diffuse type	3.41 (1.51–7.68)	0.003	3.41 (1.44–8.09)	0.005
ASA	3,4,5 vs. 1,2	1.02 (0.43–2.39)	0.968		
Approach	Open vs. MIS	0.72 (0.33–1.55)	0.395		
Procedural type	TG vs. TG [Exception]	0.90 (0.40–2.05)	0.802		
pT	pT4 vs. pT1,2,3	0.62 (0.29–1.32)	0.219		
pN	pN1,2,3 vs. N0	0.78 (0.26–2.33)	0.659		
pM	M1 vs. M0	2.68 (1.12–6.35)	0.025	3.99 (1.51–10.55)	0.005
Surgery time (min)	< 308 vs. 308 ≤	1.30 (0.62–2.75)	0.489		
Time to specimen processing (min)	< 120 vs. 120 ≤	2.63 (1.09–6.67)	0.033	2.65 (1.01–6.98)	0.049

*L* Antrum, *M* Body, *U* Upper portion, *ASA* American Society of Anesthesiologists physical status, *MIS* Minimal invasive surgery, *TG* Total gastrectomy

## Discussion

Trastuzumab in combination with chemotherapy and trastuzumab deruxtecan in tumors with HER2 high expression demonstrated a significant overall survival improvement compared with the group administered only chemotherapy, as the development of companion diagnosis and molecular-targeting therapeutic agents has progressed in recent years [13, 14]. Furthermore, trastuzumab deruxtecan has been recently revealed to show clinical activity even in GC with low HER2 expression, and the importance of more accurate HER2 expression evaluation is increasing [31]. In the present study, we demonstrated that the time to specimen processing, the intestinal type of cancers and pM are independent factors involving HER2-positivity. Previously, the time to specimen processing was associated with HER2 positivity, which was based on the results of studies using breast cancer specimens, and the relationship between GC and EGJC remained unclear. The present results indicate that time to specimen processing is an important factor in HER2-positive rates in GC and EGJC as well as in breast cancer, and it may have important prognostic implications for GC and EGJC.

In prior large-scale clinical studies, it was found that the HER2-positive rate of GC and EGJC ranges from 13.6% to 21.3% [11, 14, 17], and the results of the present study were similar at 21.2%. Furthermore, according to previous reports, it was found that HER2-positivity is higher when the histological type is the intestinal type rather than the diffuse/mixed type [8, 14, 32, 33]. In this study, we also found similar results. However, aside from a report indicating a relationship between distal metastasis and HER2-positive rate [32], as per the present study, reports have suggested that distal metastasis and staging show no correlation with HER2-positive rate [8, 17], and reports have indicated that the rate of HER2-positivity is high in patients with liver metastasis, but low in patients with peritoneal dissemination [17, 34]. This is the main reason why it remains controversial. We believe that further examination is needed in future. In breast cancer tissue, delay to formalin fixation had an adverse impact on IHC and FISH results of HER2 [21–24], and it reportedly caused poor stainability on IHC and poor diagnostic accuracy of fluorescence IHC. Similarly, in the present study, we demonstrated that specimen processing time of > 120 min has a negative impact on the HER2 positive rate.

FFPE specimens are typically used in molecular diagnosis, including HER2 diagnosis; however, diagnosis via FFPE is affected by the “preformalin fixation process” in specimen processing, including the warm ischemic time, cold ischemic time, and amount of specimen tissue; the “formalin fixation conditions” including the formalin concentration and pH, and the formalin fixation time; and the “postformalin fixation process” including the decalcification processing, and reagent and FFPE preservation conditions [21–28]. The preformalin fixation process is the only process involving clinicians (surgeons), which is important for creating high-quality FFPR specimens. The College of American Pathologists/American Society for Clinical Pathology/American Society of Clinical Oncology guidelines [4], Japanese Guidelines for HER2 Testing in Breast Cancer/Gastric Cancer [5], and the Japanese Classification of Gastric Carcinoma [29] have presented the specimen handling in detail, and for laboratories that conduct HER2 testing, these guidelines strongly recommend adopting the same tests in the program to improve the overall quality of the laboratories and to establish an appropriate monitoring system to improve quality. Our institution has created a manual for laboratory processing and pathological diagnosis using formalin fixation until diagnosis that observes these guidelines. However, the surgical time affects the warm ischemic time, while the cold ischemic time is easily affected by the processing of clinicians handling the specimen. We believe that variation in the time to specimen processing caused by these factors consequently affected the HER2-positive rate in the present study. In this study, we again demonstrated the importance of the “preformalin fixation process” involving clinicians (surgeons). In breast cancer surgery, formalin fixation of surgical specimens is performed within 1 h at approximately 50% of institutions; however, at approximately 20% of institutions, the time to formalin fixation reportedly requires more than 2 h [5]. The time until formalin fixation is expected to be longer in surgery for GC and EGJC, which has a longer surgery time than breast cancer. It is possible that reviewing the “preformalin fixation process” and reducing the time to specimen processing can reduce false-negative HER2 results. Therefore, accurate HER2 expression can be evaluated in GC and EGJC, increasing the opportunity to administer molecular-targeted therapy that can appropriately expect therapeutic effects to patients.

There are several limitations to this study. First, the time from complete resection of the stomach until the “start” of specimen processing was defined as the time to specimen processing; however, this is not strictly the time required for specimen processing. To be precise, we believe the total time, including the warm ischemic time from cessation of tissue blood flow and cold ischemic time until specimen fixation after resection, should be calculated. Moreover, a small difference was observed in the time required for specimen processing in each case, and we believe the effect was small. Second, the HER2 expression rate is reportedly affected by the “formalin fixation conditions,” such as the formalin concentration and pH, as well as the time taken and conditions of formalin fixation of the specimen [25]; however, these factors were not examined in the present study. Yet, the present study is a single-center study, and the processing from formalin fixation until diagnosis was compiled into a manual, which did not change during the study period. Therefore, regarding the “formalin fixation conditions,” we believe there was little impact of this limitation on the cases. Third, HER-2 heterogeneity in GC has been confirmed within the same tumor, and even within the same tumor gland; thus, HER-2 expression should be tested with at least 3–4 slides in tumors other than HER2 3 + [35, 36]. This study tested HER2 expression with only one slide. Therefore, some cases judged as false negatives for HER2 expression may be included in the group we analyzed. Additionally, we excluded metastatic lesions from the analysis and evaluated HER-2 expression only in GC primary lesions to prevent bias, but HER2 expression generally differs between primary and metastatic lesions, and verification, including metastatic lesions, may be necessary [37]. Fourth, this is a single-center retrospective study with a small subject sample; therefore, a multicenter collaborative prospective study is necessary.

## Conclusion

In the present study, we demonstrated that in gastric cancer and esophagogastric junction cancer, intestinal type, pM, and time to specimen processing of < 120 min were independent factors involving HER2-positivity. By improving the preformalin fixation process and reducing the tine to specimen processing, we believe that the risk of false-negative HER2 results can be reduced.

## Data Availability

Data will be made available by the corresponding author upon reasonable request.

## Abbreviations

CI: Confidence interval
EGJC: Esophagogastric junction cancer
FFPE: Formalin-fixed paraffin-embedded
FISH: Fluorescence in situ hybridization
GC: Gastric cancer
HER2: Human epidermal growth factor receptor 2
IHC: Immunohistochemistry
OR: Odds ratio
